# Mavacamten in Obstructive Hypertrophic Cardiomyopathy and C282Y Homozygous Hereditary Hemochromatosis: A First-in-Human Report

**DOI:** 10.1016/j.jaccas.2026.107736

**Published:** 2026-03-31

**Authors:** Riya Sam, Anil Ananthaneni, Maria Isabel Planek, Alan Kogan, Robert Gordon

**Affiliations:** a Division of Cardiology, Endeavor Health, Glenview, Illinois, USA; b Division of Cardiology, University of Chicago Pritzker School of Medicine, Chicago, Illinois, USA; c Division of Hematology/Oncology, Louisiana State University Health Sciences, Shreveport, Louisiana, USA

**Keywords:** cardiac myosin inhibitors, hereditary hemochromatosis, hypertrophic cardiomyopathy, mavacamten, multimodality imaging

## Abstract

**Background:**

Obstructive hypertrophic cardiomyopathy (HCM) and hereditary hemochromatosis (HH) rarely coexist. The therapeutic role of cardiac myosin inhibitors in this setting has not been described.

**Case Summary:**

A 67-year-old woman with known homozygous C282Y HH was referred for an abnormal stress echocardiogram. Cardiac magnetic resonance showed septal hypertrophy, systolic anterior motion of the mitral valve with left ventricular outflow tract obstruction, and iron overload. Owing to beta-blocker intolerance, mavacamten was started, with marked reduction in left ventricular outflow tract gradient.

**Discussion:**

This case highlights the complexity of diagnosing and managing obstructive HCM in the context of HH. To our knowledge, this is the first reported use of mavacamten in this setting, demonstrating a favorable hemodynamic and clinical response.

**Take-Home Messages:**

Multimodality imaging is essential to differentiate primary obstructive HCM from HH-related cardiomyopathy. In this case, mavacamten was a well-tolerated alternative, though further data are needed to establish broader applicability.

## Visual Summary

**Figure:**
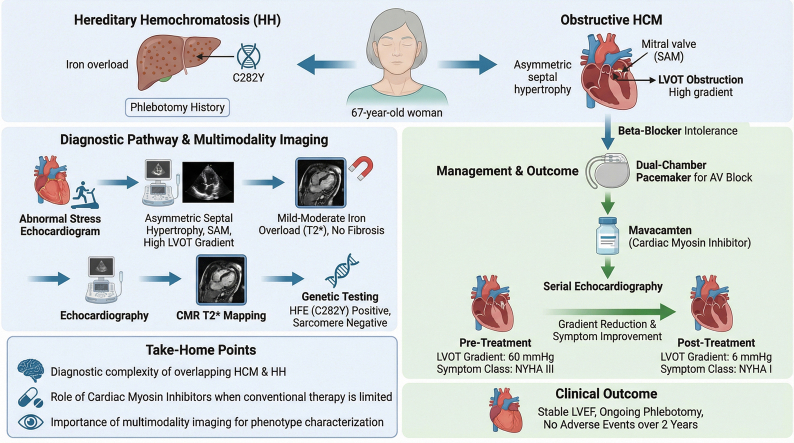

## History of Presenting Illness

A 67-year-old woman was referred to the outpatient cardiology clinic after an abnormal stress echocardiogram performed as part of preprocedural evaluation for a routine colonoscopy under general anesthesia. She had progressive exertional dyspnea and lightheadedness associated with chest heaviness over a period of 6 months, which prompted her primary care physician to order the test. Physical examination was notable for a grade III crescendo-decrescendo systolic murmur at the precordium that increased with the Valsalva maneuver, reproducing her symptoms of lightheadedness.

**Take-Home Messages**

• To recognize the diagnostic challenges in differentiating primary obstructive hypertrophic cardiomyopathy from cardiomyopathy secondary to hereditary hemochromatosis.
• To use multimodality imaging to characterize complex, overlapping cardiomyopathic phenotypes.
• To evaluate the role of cardiac myosin inhibitors as a safe therapeutic option when conventional medical therapy is limited by intolerance.

## Past Medical History

The patient had been diagnosed with hereditary hemochromatosis (HH) 10 years earlier on evaluation of elevated liver enzymes. Genetic testing was homozygous for C282Y mutation. Since then, she had been undergoing therapeutic phlebotomy—initially 4 times a year, and then eventually monthly per her treating hematologist, guided by iron studies. She had no family history of sudden cardiac death (SCD), hypertrophic cardiomyopathy (HCM), or hemochromatosis. Notably, she had no prior cardiology evaluations or cardiac imaging before this referral. Her only other medication was levothyroxine for hypothyroidism.

## Differential Diagnosis

The differential diagnosis included aortic stenosis, coronary artery disease, iron overload cardiomyopathy, dynamic left ventricular outflow tract (LVOT) obstruction secondary to loading conditions, and HCM.

## Investigations

**Figure 1:**
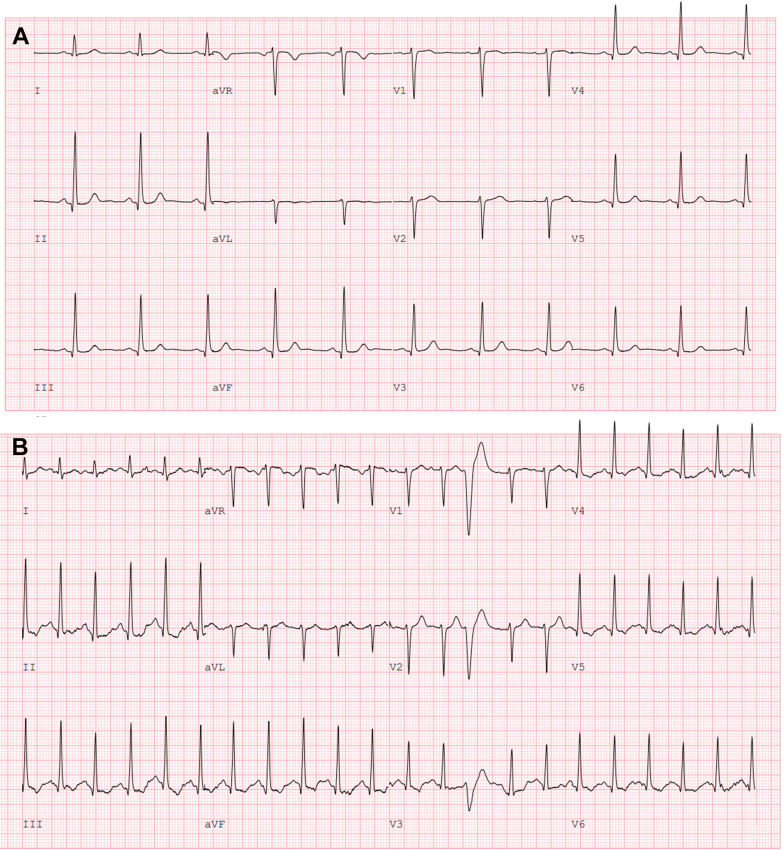
ECG at Baseline and Peak Stress (A) Baseline ECG showing normal sinus rhythm and no evidence of left ventricular hypertrophy. (B) ECG at peak stress, with no definitive evidence of exercise-induced ischemia or conduction disease. ECG = electrocardiogram.

Laboratory testing showed a hemoglobin of 13 g/dL, mean corpuscular volume 101 fL, mean corpuscular hemoglobin 33.5 pg, and serum ferritin 61 ng/mL. Baseline electrocardiogram showed a normal sinus rhythm without voltage criteria for left ventricular hypertrophy (Figure 1A). During the stress test obtained before referral, she was able to walk for 4 minutes on the Bruce protocol and attained a peak heart rate of 126 beats/min before stopping due to dyspnea and chest discomfort. There was no electrocardiographic evidence of exercise-induced ischemia or advanced conduction abnormalities (Figure 1B). Stress echocardiogram was notable for evidence of systolic anterior motion (SAM) and mitral regurgitation (MR), with an elevated LVOT gradient of 27 mm Hg post-exercise, without ischemia at peak stress.

**Figure 2:**
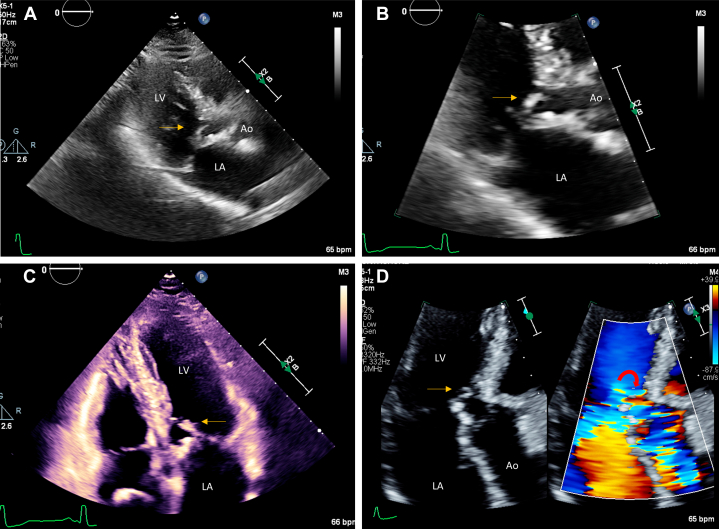
Findings on Initial Transthoracic Echocardiography (A) Parasternal long-axis view and (B) zoomed-in parasternal long-axis view with calcified anterior mitral valve leaflet with systolic anterior motion (yellow arrow). (C) Apical 5-chamber view with systolic anterior motion (yellow arrow) and (D) Apical 3-chamber view with systolic anterior motion (yellow arrow) and resultant posteriorly directed mitral regurgitation and flow acceleration through the LVOT on color Doppler (red curved arrow). Ao = aorta; LA = left atrium; LV = left ventricle; LVOT = left ventricular outflow tract.

**Figure 3:**
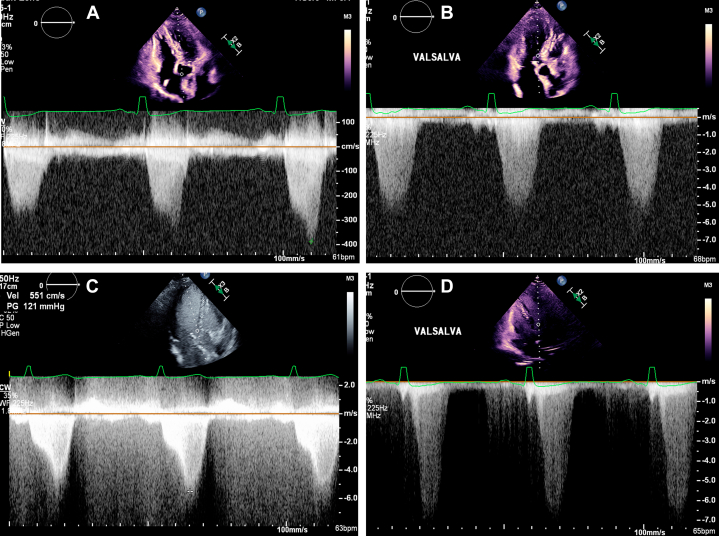
LVOT Gradients at Rest and With Valsalva Maneuver LVOT continuous-wave Doppler (A) at rest and (B) during Valsalva. LVOT contrast-enhanced continuous-wave Doppler (C) at rest and (D) during Valsalva. The maximum provoked gradient was 170 mm Hg. LVOT = left ventricular outflow tract.

**Figure 4:**
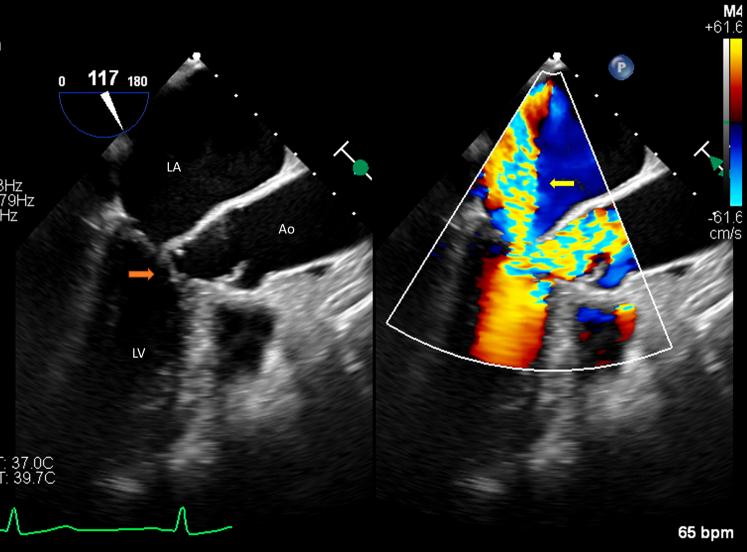
Transesophageal Echocardiography of the Mitral Valve Long-axis midesophageal view of the mitral valve at ∼120° demonstrating systolic anterior motion (red arrow) with resultant moderate to severe posteriorly directed mitral regurgitation on color Doppler (yellow arrow).

A dedicated structural transthoracic echocardiogram confirmed asymmetric basal septal hypertrophy with a maximal wall thickness of 15 mm, a normal left ventricular ejection fraction (LVEF) of 72%, normal diastolic function, and normal global longitudinal strain. Aortic valve was trileaflet, with no stenosis and mild aortic regurgitation. The anterior mitral valve leaflet was calcified with SAM, resulting in moderate to severe posteriorly directed MR (Video 1, Figure 2). The resting LVOT gradient was 60 mm Hg, and the maximum provoked contrast-enhanced gradient with Valsalva maneuver was 170 mm Hg (Figure 3). Genetic testing for sarcomeric and other familial cardiomyopathies was negative. Repeat *HFE* testing confirmed a pathogenic homozygous C282Y mutation. Transesophageal echocardiography demonstrated a calcified anterior mitral valve leaflet, SAM of the leaflet and subvalvular apparatus, and moderate to severe posteriorly directed MR (Video 2, Figure 4). The LVOT gradient under sedation was 35 mm Hg. Coronary computed tomography angiography showed no evidence of coronary atherosclerosis, with an Agatston calcium score of zero.

**Figure 5:**
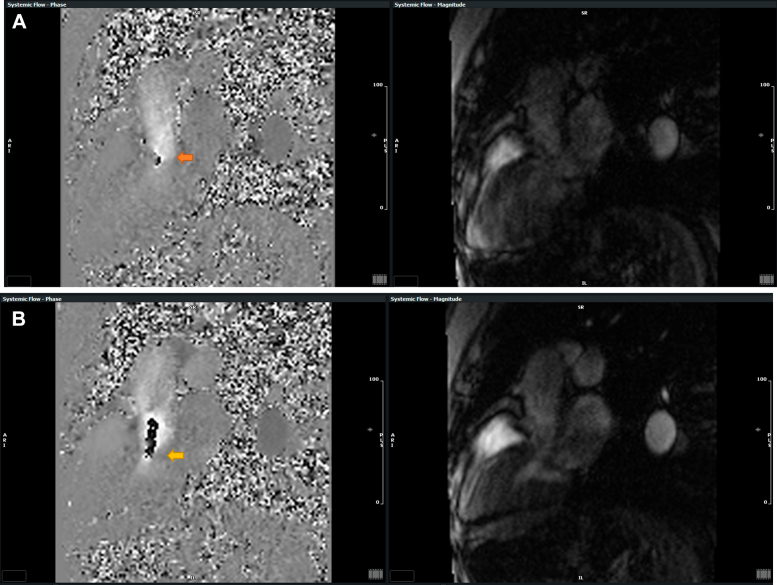
CMR Phase-Contrast Image of Apical 3-Chamber/LVOT View Showing the LVOT Obstruction Associated With the Systolic Anterior Motion The phase-contrast image is on the left, the magnitude image is on the right. (A) At a velocity encoding of 3.7 m/s with a resting gradient of at least 55 mm Hg (red arrow). (B) At a velocity encoding of 2.5 m/s, showing the difference in aliasing (yellow arrow). CMR = cardiac magnetic resonance; LVOT = left ventricular outflow tract.

**Figure 6:**
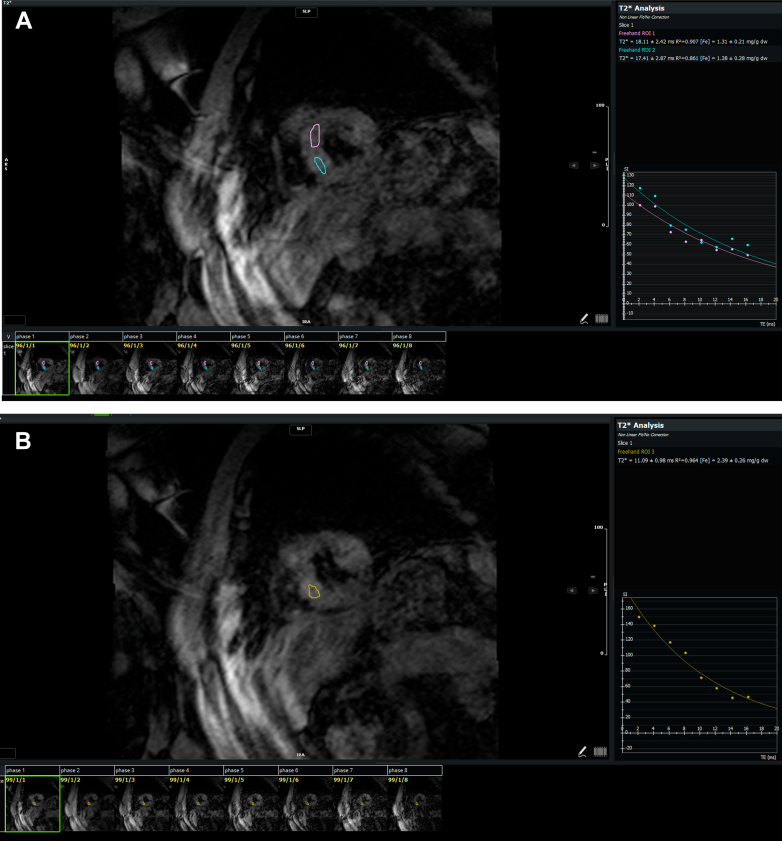
CMR T2∗ Images of the Basal and Mid Septum (A) Basal short-axis septum and (B) mid short-axis septum demonstrating abnormally low T2∗ times (<20 ms), suggestive of mild to moderate iron overload. CMR = cardiac magnetic resonance.

Cardiac magnetic resonance revealed normal biventricular function and mild aortic regurgitation. There was asymmetric basal septal hypertrophy measuring 13 mm in the basal anteroseptal segment and 7 mm in the basal inferolateral segment. The anterior mitral valve leaflet was calcified and elongated, with prominent leaflet and chordal SAM and dynamic LVOT obstruction, with a maximum resting gradient of at least 55 mm Hg (Video 3, Figure 5). There was mild to moderate posteriorly directed MR, a left atrial area of 28 cm^2^, left ventricular mass of 105 g, an indexed mass of 54 g/m^2^ (normal: <75 g/m^2^), and a left ventricular end-diastolic volume index of 68 mL/m^2^ (normal: <66 mL/m^2^). Decreased T2∗ times (<20 ms) in the basal and mid septum were consistent with mild to moderate cardiac iron overload (Figure 6). There was hypertrophy of the papillary muscle and suggestion of a crypt in the basal-to–mid inferoseptal segment. Extracellular volume was normal at 26% at a hematocrit of 39%, and there was no evidence of abnormal late gadolinium enhancement to suggest focal myocardial fibrosis or scar. Other anatomic markers of HCM were absent.

## Management

**Figure 7:**
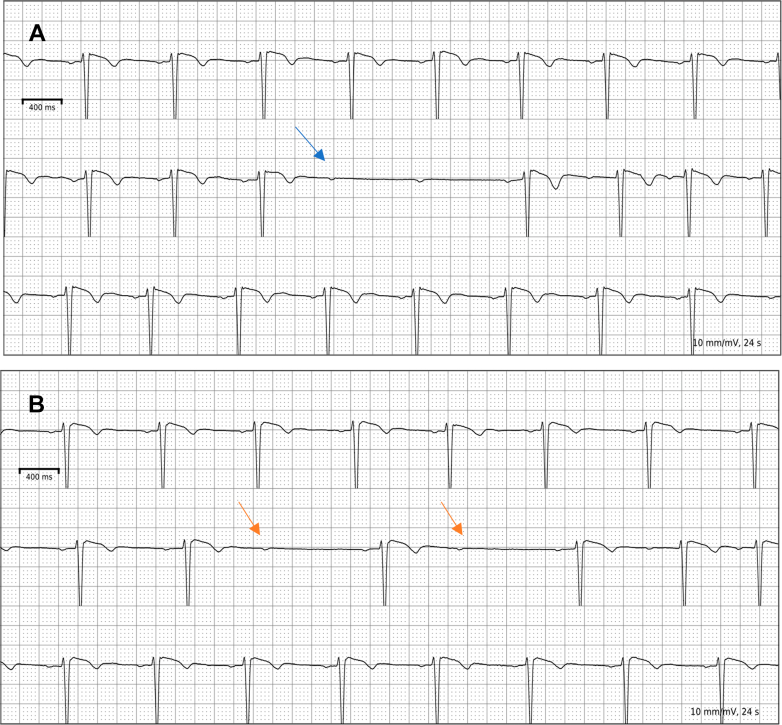
Findings on Outpatient 14-Day Ambulatory Monitor (A) Outpatient rhythm monitor with evidence of high-grade atrioventricular block (blue arrow) in the setting of syncope. (B) There were a few occurrences of Mobitz type I atrioventricular block during the monitoring (red arrow).

**Figure 8:**
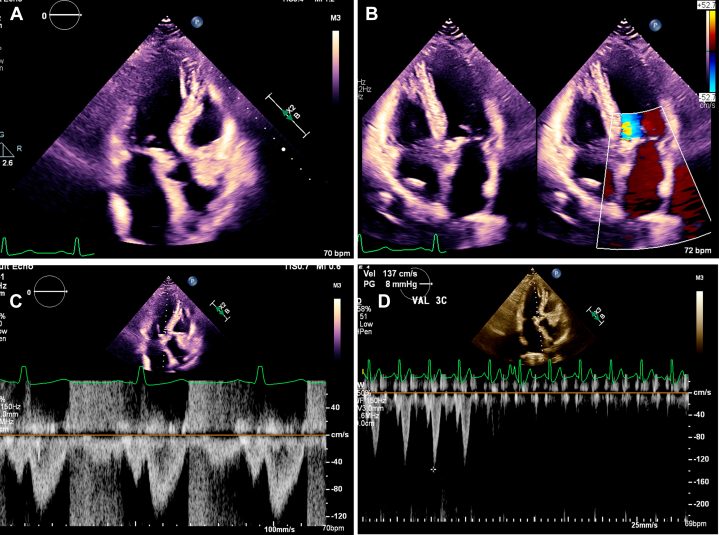
TTE After 12 Months of Mavacamten Therapy Demonstrating Stable and Decreased LVOT Gradients and a Reduction in MR (A) Apical 3-chamber view with the degree of systolic anterior motion reduced compared to prior. (B) Apical 4-chamber view with no significant MR. (C) LVOT resting pulsed-wave Doppler without aliasing and a peak velocity of 1.2 m/s (D) LVOT pulsed-wave Doppler during Valsalva maneuver with a gradient of 8 mm Hg and maneuver release showing a decrease in the velocity. LVOT = left ventricular outflow tract; MR = mitral regurgitation; TTE = transthoracic echocardiography.

Although the contribution of HH to the phenotype remained uncertain, the patient had clear evidence of severe obstructive HCM. She was started on metoprolol succinate 50 mg daily and subsequently increased to 100 mg daily, which she did not tolerate given profound fatigue. A 14-day ambulatory monitor captured high-grade atrioventricular block during an episode of syncope (Figure 7). Based on the American Heart Association HCM SCD risk score (2%), there was no definitive indication for an implantable cardioverter-defibrillator. Following discussion with electrophysiology, she underwent dual-chamber pacemaker implantation. Given persistent symptoms (NYHA functional class III) and high provoked gradients, a multidisciplinary heart team discussed medical therapy, and the decision was made to initiate mavacamten 5 mg daily. Adherence to the REMS (Risk Evaluation and Mitigation Strategy) program was strictly maintained, including serial echocardiography and monitoring for drug-drug interactions (CYP2C19/CYP3A4). The patient continued to have residual exertional symptoms, with a provoked gradient of >70 mm Hg and normal LVEF, and so the dose was increased to 10 mg by month 3. Follow-up echocardiograms showed a significant reduction in provoked gradients, which subsequently stabilized, and her symptoms gradually improved (Video 4, Figure 8).

## Outcome and Follow-Up

**Figure 9:**
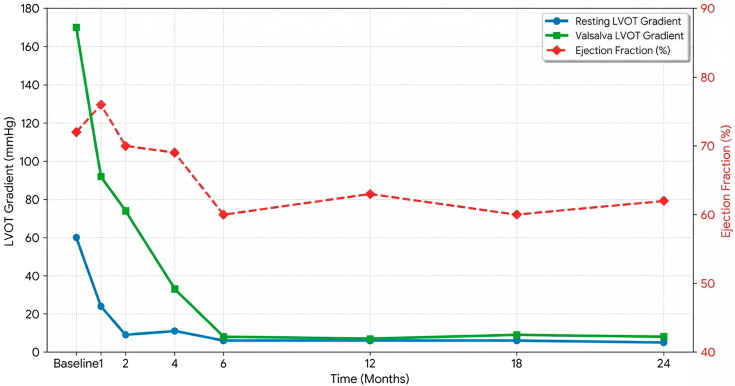
Longitudinal Hemodynamic Response to Mavacamten Trend of left ventricular ejection fraction (red), provoked Valsalva LVOT gradient (green), and resting LVOT gradient (blue) over 24 months. LVOT = left ventricular outflow tract.

Serial echocardiographic monitoring demonstrated a marked reduction in LVOT gradient, with the most recent values being 6 mm Hg at rest and 8 mm Hg with Valsalva, as well as SAM with mild MR, 2 years after the initiation of treatment (Figure 9). LVEF remained stable at >50% with continued monthly phlebotomy. She was able to return to her daily activities and reported significant improvement in her symptoms.

## Discussion

HCM is defined by left ventricular hypertrophy that cannot be explained solely by abnormal loading conditions or myocardial infiltration.^1^ Obstructive HCM represents a major subgroup in which dynamic LVOT obstruction, typically caused by basal septal hypertrophy and SAM of the mitral valve, leads to severe exertional symptoms.^2^ As the most common genetic cardiomyopathy (prevalence ∼1:500), it is a leading cause of heart failure and SCD.^2^

Management of obstructive HCM has traditionally relied on negative inotropes, such as beta-blockers or nondihydropyridine calcium-channel blockers. However, these agents do not target the underlying sarcomeric hypercontractility and may be limited by side effects or conduction disease. The 2024 American Heart Association/American College of Cardiology guidelines now provide a Class I recommendation for cardiac myosin inhibitors in patients with persistent symptoms despite first-line medical therapy.^1^ Clinical trials, including EXPLORER-HCM and VALOR-HCM, established the efficacy of mavacamten in reducing gradients and improving NYHA functional class.^3^^,^^4^ Furthermore, the MAPLE-HCM trial recently demonstrated that the myosin inhibitor aficamten was superior to metoprolol monotherapy in improving exercise capacity (peak Vo_2_) and health status.^5^

HH is an autosomal recessive disorder of iron metabolism, most frequently caused by C282Y homozygosity. Cardiac iron deposition typically manifests as restrictive or dilated cardiomyopathy.^2^ While hypertrophic phenotypes in HH are rare and usually attributed to secondary fibrosis, the classic asymmetric hypertrophy and SAM seen in this patient are atypical for isolated HH.^6^^,^^7^ Management relies on therapeutic phlebotomy to reduce total body iron, which has been shown to improve cardiac hemodynamics in iron-overload states.^8^

The clinical scenario of this patient was uniquely challenging given the coexistence of 2 distinct pathologies. Multimodality imaging was critical: Echocardiography confirmed the obstructive mechanism, while cardiac magnetic resonance T2∗ mapping quantified the myocardial iron burden. The absence of sarcomere mutations alongside *HFE* homozygosity suggests a complex phenotype where iron-mediated stress and primary hypertrophic drivers may independently contribute to the hyperdynamic, obstructive physiology.

The overlap of obstructive HCM and HH is rare and has not been described in the literature, and the role of cardiac myosin inhibition in this dual pathology remains undefined. In the present case, mavacamten provided a safe and effective mechanism to address the hypercontractility without exacerbating the vulnerabilities often present in iron-overload cardiomyopathy.

## Conclusions

To the best of our knowledge, this is the first reported use of a cardiac myosin inhibitor in C282Y homozygous HH with obstructive HCM, demonstrating significant LVOT gradient reduction, improvement in NYHA functional class from III to I, stable LVEF under ongoing phlebotomy, and no safety signal over 24 months. Randomized clinical trials for this population are unlikely to be feasible. While this case illustrates a successful application of targeted sarcomere therapy in a complex phenotype, further data are required to establish the safety of cardiac myosin inhibitors in mixed cardiomyopathy populations.

**Visual Summary:**
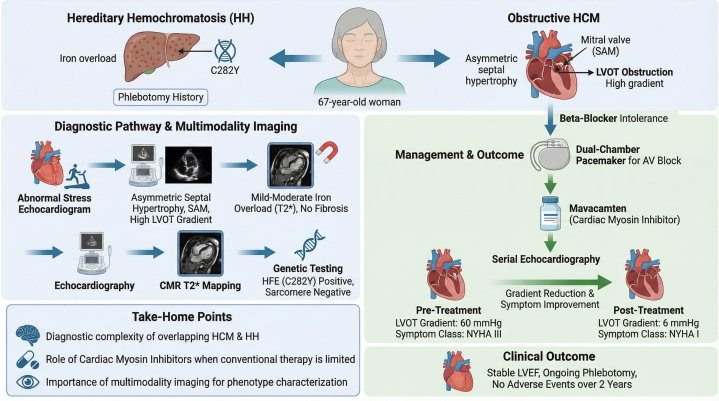
Cardiac Myosin Inhibition in Obstructive Hypertrophic Cardiomyopathy and Hereditary Hemochromatosis AV = atrioventricular; CMR = cardiac magnetic resonance; HCM = hypertrophic cardiomyopathy; LVEF = left ventricular ejection fraction; LVOT = left ventricular outflow tract; SAM = systolic anterior motion.

## Funding Support and Author Disclosures

The authors have reported that they have no relationships relevant to the contents of this paper to disclose.

## Appendix

Video 1 Transthoracic Echocardiography Apical 3-Chamber Zoomed-In View Showing SAM With Posteriorly Directed Moderate Mitral Regurgitation, Mild Aortic Regurgitation, and LVOT Flow Acceleration

Video 2 Transesophageal Echocardiography Long-Axis Midesophageal View of Mitral Valve at ∼120° Demonstrating Calcified Anterior Mitral Valve Leaflet With SAM and Moderate to Severe Posteriorly Directed Mitral Regurgitation and Mild Aortic Regurgitation

Video 3 Steady-State Free Precession Cardiac Magnetic Resonance Cine Imaging Demonstrating Left Ventricular Hypertrophy and SAM of the Mitral Valve

Video 4 Transthoracic Echocardiography Apical 3-Chamber Views Showing SAM With Mild Mitral Regurgitation and Mild Aortic Regurgitation
